# A longitudinal analysis of population-level lipid and apolipoprotein trends over two decades: descriptive assessment using patient medians in a Swedish tertiary care center

**DOI:** 10.1186/s12944-025-02830-0

**Published:** 2025-12-12

**Authors:** Kristina Sejersen, Anders O. Larsson

**Affiliations:** 1 Department of Medical Sciences, Uppsala University, Uppsala University Hospital, entrance 61, Uppsala, 75185 Sweden; 2 Unilabs AB, Stockholm, 17154 Sweden

**Keywords:** Cardiovascular diseases, Lipids, Lipoproteins, LDL-C, HDL-C, ApoB, ApoA1, Clinical laboratory techniques, Quality control, Population surveillance

## Abstract

**Background:**

Cardiovascular disease (CVD) remains the leading cause of mortality worldwide. Lipid biomarkers, including direct low-density lipoprotein cholesterol (LDL-C), high-density lipoprotein cholesterol (HDL-C), apolipoprotein B (ApoB), and apolipoprotein A1 (ApoA1), are essential tools for cardiovascular risk assessment. Monitoring patient-derived median values over time may provide insights into population health and analytical performance. This study provides a descriptive analysis of population-level lipid results spanning nearly two decades. While trends in patient medians may support quality assurance, these data do not constitute a validated approach to risk prediction or definitive analytical monitoring due to the absence of outcome and treatment information.

**Methods:**

We retrospectively analyzed routine clinical laboratory data from Uppsala University Hospital, Sweden, covering January 2006–December 2024. A total of 890,948 LDL-C, 867,446 HDL-C, 64,787 ApoB, and 65,500 ApoA1 results were included. Measurements were performed on Abbott Architect systems until 2021, after which assays were transferred to Roche Cobas Pro platforms. Statistical analyses included trend evaluation, variability assessment, and seasonal pattern analysis.

**Results:**

Women had modestly higher LDL-C and HDL-C levels compared to men, while ApoB values were similar between sexes. ApoA1 was notably higher in women. Over the 19-year period, median LDL-C declined from 3.18 to 2.62 mmol/L, consistent with improved lipid management. HDL-C remained stable (1.36–1.45 mmol/L), while ApoB and ApoA1 concentrations showed minimal change. Variability was highest for LDL-C (median CV 6.4%) and lowest for ApoA1 (median CV 2.6%). Seasonal variation was negligible across all analytes. Testing volumes increased substantially for LDL-C and HDL-C, whereas ApoB and ApoA1 requests peaked around 2010 and later declined.

**Conclusions:**

Long-term monitoring of median patient values demonstrates declining LDL-C, stable HDL-C, and consistent ApoB/ApoA1 ratios with minimal seasonal effects. These findings highlight the potential utility of patient medians as supplementary quality indicators and for population-level lipid surveillance.

## Background

Cardiovascular disease (CVD) remains the leading cause of mortality in Europe and globally [1]. Its predominant manifestation is atherosclerotic cardiovascular disease (ASCVD). This underscores the continuous need for accurate risk assessment, early diagnosis, and targeted prevention strategies. In Europe alone, CVD accounts for over 4 million deaths each year. Women bear a greater absolute burden, with 2.2 million deaths compared to 1.8 million in men. However, premature CVD-related mortality, defined as death before the age of 65, is more prevalent among men, with approximately 490,000 cases versus 193,000 among women [2].

Lipid biomarkers, particularly low-density lipoprotein cholesterol (LDL-C) and high-density lipoprotein cholesterol (HDL-C), are among the most important clinically used tools for cardiovascular risk evaluation. LDL-C refers to the cholesterol content within low-density lipoprotein (LDL) particles rather than the particles themselves. Elevated LDL-C plays a central role in the development of ASCVD. There is robust clinical evidence showing that lowering LDL-C significantly reduces the risk of major cardiovascular events. In contrast, high-density lipoprotein (HDL) is involved in reverse cholesterol transport and is generally considered protective. However, HDL-C is not a primary therapeutic target in clinical guidelines [3].

Apolipoproteins provide additional value for risk stratification. Apolipoprotein B (apoB), the primary protein component of atherogenic lipoproteins, provides a direct measure of particle number. ApoB may outperform traditional lipid markers, particularly in individuals with high triglyceride levels. Apolipoprotein A1 (ApoA1), the primary protein in HDL particles, reflects HDL quantity and function; however, its routine clinical utility remains limited [4, 5].

Standard lipid panels usually include total cholesterol, LDL-C, HDL-C, and triglycerides. LDL-C is most commonly estimated using the Friedewald formula. However, this method becomes less accurate at higher triglyceride concentrations. Several alternative equations have been proposed to improve estimation across a wider range of triglyceride levels. These include the Martin/Hopkins Eqs [6–8]., the Sampson Eq [9]., and the more recent Modified Sampson Method, which is designed to enhance accuracy, particularly in samples with moderate to severe hypertriglyceridemia [10].Additional markers, such as non-HDL-C and apoB, are increasingly used for more precise risk assessment, especially in patients with metabolic disturbances [3, 5]. Non-HDL-C and ApoB are particularly informative in patients with hypertriglyceridemia, mixed dyslipidemia, type 2 diabetes, or metabolic syndrome, and they are also useful for identifying phenotypes such as type III hyperlipoproteinemia or high‑risk normotriglyceridemic hyperapoB individuals [11–14].

Because lipid biomarkers play a central role in guiding clinical decision-making, it is important to ensure long-term consistency in the measurements available to clinicians [11, 12, 15]. Traditional quality control procedures are designed to detect acute analytical errors but may not capture gradual shifts or long-term trends in patient results. While our study does not directly assess analytical performance, such as by repeatedly testing the same samples over time, it does evaluate trends in population-based median patient values. These values can serve as a complementary indicator for identifying potential systematic changes at the population level [13]. Although median patient lipid values may complement laboratory quality assessment, they cannot independently identify analytical changes or support clinical risk stratification without linked outcome and medication data. Our findings should be interpreted as descriptive, hypothesis-generating insights into routine lipid surveillance.The present study aimed to evaluate the usefulness of median patient values for LDL-C, HDL-C, ApoB, and ApoA1 in monitoring analytical performance over time and to assess their potential as supplementary tools for improving the reliability of lipid measurements in clinical laboratories.

## Materials and methods

### Samples

Routine requests for LDL-C, HDL-C, ApoB, and ApoA1 were obtained from the Department of Clinical Chemistry at Uppsala University Hospital, a large academic tertiary care center in central Sweden that serves local patients and regional referrals. These samples were collected using plasma tubes (367962, BD Vacutainer Systems, Plymouth, UK). Patients were instructed to fast overnight when appropriate. However, both fasting and non-fasting samples were included according to clinical routine. Plasma was separated within five hours of sampling by centrifugation at 1,300–2,400 × g for five to ten minutes. After separation, the samples were stored at 2–8 °C for up to six days and analyzed without freezing them beforehand. EDTA (ethylenediaminetetraacetic acid) plasma was not used due to known analytical interference. The exact fasting status was not documented for all patients, which is a limitation of the study. The study period spanned from January 2006 to December 2024. Data were extracted without full patient identifiers in agreement with the ethical permit. The following information was included: sampling month and year, age (in years), sex, and LDL-C, HDL-C, ApoB, and ApoA1 values.

### Laboratory methods

All lipid and apolipoprotein assays were conducted in an ISO 15,189-accredited laboratory environment. Calibration was performed using reference materials that are traceable to international standards: IFCC/WHO for LDL-C and ApoB and CDC/CRMLN for HDL-C. Method changes and transitions between platforms included systematic cross-validation with parallel testing of clinical samples and third-party quality panels. Cross-platform comparability was verified using regression statistics and bias analyses. All procedures were performed in accordance with written protocols for accredited clinical laboratories in Sweden.

Throughout the study period, plasma or serum concentrations of LDL-C, HDL-C, ApoB, and ApoA1) were analyzed using standardized laboratory protocols. The laboratory’s analytical performance for these analytes was monitored by participating in EQUALIS proficiency testing for both serum and plasma matrices. Blinded external quality assessment surveys were conducted four times a year. Results consistently fell within official analytical acceptance limits, showing no detectable bias or drift across analytical platforms. Internal quality control (QC) was performed daily for all lipid and apolipoprotein assays using manufacturer-recommended control materials and Westgard-type rules to ensure ongoing analytical stability. Initially, all assays were performed on an Architect c8000 chemistry analyzer (Abbott Laboratories, Abbott Park, IL). LDL-C was measured using reagent 1E31-20 (Abbott Laboratories). HDL-C analysis used reagent 3K33-20 until 2010; then, reagent 3K33-21 (Abbott Laboratories) was employed. ApoB was assayed using reagent 9D92-01 until 2010 and then reagent 9D93-21. ApoA1 was measured using reagent 9D92-01 until 2010; then, reagent 9D92-20 (Abbott Laboratories) was introduced. In 2021, all lipid and apolipoprotein analyses were transferred to a Cobas Pro analyzer (Roche Diagnostics, Rotkreuz, Switzerland). LDL-C was measured using the LDLC3 kit (art. No. 8057966190, Roche). HDL-C analysis used HDL-Cholesterol Gen. 4 (art. No. 8057877190, Roche). ApoB was determined using APOBT, Tina-quant Apolipoprotein B version 2 (art. No. 8105464190), and ApoA1 with APOA1T, Tina-quant Apolipoprotein A-1, version 2 (art. No. 8105448190). 2 (art. No. 8105448190, Roche). Method verification was conducted when transitioning from the Abbott system to the Roche system. Linear regression analysis of patient samples yielded the following relationships: For LDL-C, Cobas LDL-C = 0.98 × Architect LDL-C + 0.12, with Roche values averaging 1.3% lower than Abbott values. For HDL-C, Cobas HDL-C = 1.08 × Architect HDL-C – 0.05, with Roche measurements approximately 7.7% higher. For ApoB, Cobas ApoB = 0.98 × Architect ApoB + 0.01, showing no systematic bias and Roche values averaging 1% lower. For ApoA1, Cobas ApoA1 = 0.96 × Architect ApoA1 + 0.03, with Roche values approximately 6% lower than Abbott values in patient samples.

### Statistical analyses

Statistical analyses, including linear regression and correlation coefficient calculations, were performed using Excel 365 (Microsoft Corporation, Seattle, WA, USA) and Statistica 10 (Tibco Software, Palo Alto, CA, USA). Data are presented as the median, lower quartile, upper quartile, 10th percentile, and 90th percentile. Categorical variables are expressed as the number of cases (% of total). The coefficients of variation (CVs) for LDL-C, HDL-C, ApoB, and ApoA1 were calculated using annual, population-level summary statistics (median, quartiles, and percentiles) over the study period. Thus, they reflect the temporal variability of these aggregated measures rather than the biological variation within individuals or the analytical QC performance.

## Results

### Baseline characteristics

From January 1, 2004, to December 31, 2024, a total of 890,948 LDL-C, 867,446 HDL-C, 64,787 ApoB, and 65,500 ApoA1 measurements were reported. During the same visit, ApoB was requested with LDL-C in 31,960 panels (3.6%), and ApoA1 was requested with HDL-C in 32,442 panels (3.7%).

Among male patients, LDL-C results were obtained for 463,489 patients (52.0% of total study population), and HDL-C results were obtained for 447,752 patients (51.6%). Among female patients, LDL-C results were obtained for 427,459 patients (48.0%), and HDL-C results were obtained for 419,694 patients (48.4%). The median age of male patients with LDL-C measurements was 65 years (IQR: 54–73), and the median age of female patients was 65 years (IQR: 54–74). For HDL-C, the median age was 64 years (IQR: 53–72) for males and 64 years (IQR: 53–73) for females.

A total of 64,787 ApoB and 65,500 ApoA1 measurements were reported during the same period. Of these, 48.7% of the ApoB results and 48.6% of the ApoA1 results were recorded for male patients, while 51.3% of the ApoB results and 51.4% of the ApoA1 results were recorded for female patients. The median age of male patients with ApoB and ApoA1 measurements was 57 years (IQR 44–66) and 55 years (IQR 41–65) for females. Table 1 summarizes the age distributions, stratified by sex and analysis type.

**Table 1:** Age distributions by sex and analysis type

Analysis	Sex	Valid *N* (%)	Mean Age (years)	Median Age (years)	Lower Quartile (years)	Upper Quartile (years)	10th percentile	90th percentile	Standard deviation
LDL-C	All	890,948	62	65	54	74	41	80	16
	Men	463,489 (52.0%)	62	65	54	73	42	79	15
	Women	427,459 (48.0%)	62	65	54	74	41	80	16
HDL-C	All	867,446	61	64	53	73	40	79	16
	Men	447,752 (51.6%)	61	64	53	72	41	79	16
	Women	419,694 (48.4%)	61	64	53	73	40	80	15
ApoB	All	64,787	53	56	43	66	28	74	17
	Men	31,536 (48.7%)	54	57	44	66	28	74	17
	Women	33,249 (51.3%)	53	55	41	65	28	74	17
ApoA1	All	65,500	53	56	43	66	28	74	17
	Men	31,806 (48.6%)	54	57	44	66	28	74	17
	Women	33,692 (51.4%)	53	55	41	65	28	74	17

The median LDL-C concentration was 2.81 mmol/L (interquartile range [IQR] 2.09–3.61) in males and 3.09 mmol/L (IQR 2.39–3.89) in females. These results suggest that females had modestly higher LDL-C levels than males within the study cohort. The median HDL-C concentration was 1.17 mmol/L (IQR 0.99–1.41) in males and 1.45 mmol/L (IQR 0.70–1.40) in females. This illustrates a notable sex-related difference, with higher HDL-C observed in females.

Median ApoB concentrations were similar between the sexes: 0.93 g/L (IQR 0.76–1.12) for males and 0.92 g/L (IQR 0.76–1.11) for females. This suggests a minimal sex-related difference in ApoB levels. However, the median ApoA1 concentration was higher in females (1.54 g/L, IQR 1.35–1.77) than in males (1.38 g/L, IQR 1.22–1.56). This finding mirrors the difference in HDL-C levels, as ApoA1 is a major component of HDL particles. From an analytical perspective, the higher LDL-C and HDL-C concentrations observed in females may reflect sex-specific lipid metabolism, as often reported in the literature. The overlap of the interquartile ranges for ApoB indicates minor differences between the sexes for this marker. In contrast, the difference for ApoA1 appears more pronounced, which is consistent with the observed difference in HDL-C. Table 2 provides descriptive statistics of lipid and apolipoprotein measurements (mmol/L and g/L).

**Table 2:** Lipid and Apolipoprotein levels by group and analysis type

Analysis (units)	Group	Valid *N* (%)	Mean	Median	Lower Quartile	Upper Quartile	10th percentile	90th percentile	Standard deviation
LDL-C (mmol/L)	All	890,948	3.04	2.95	2.23	3.75	1.69	4.49	1.09
	Men	463,489 (52.0%)	2.90	2.81	2.09	3.61	1.57	4.34	1.08
	Women	427,459 (48.0%)	3.19	3.09	2.39	3.89	1.85	4.63	1.09
HDL-C (mmol/L)	All	867,446	1.36	1.30	1.07	1.59	0.90	1.90	0.41
	Men	447,752 (51.6%)	1.22	1.17	0.99	1.41	0.85	1.67	0.34
	Women	419,694 (48.4%)	1.50	1.45	1.20	1.75	1.01	2.06	0.42
ApoB (g/L)	All	64,787	0.95	0.93	0.76	1.11	0.63	1.31	0.28
	Men	31,536 (48.7%)	0.96	0.93	0.76	1.12	0.63	1.31	0.29
	Women	33,249 (51.3%)	0.95	0.92	0.76	1.11	0.63	1.31	0.28
ApoA1 (g/L)	All	65,500	1.49	1.46	1.27	1.67	1.13	1.90	0.30
	Men	31,806 (48.6%)	1.40	1.38	1.22	1.56	1.09	1.75	0.27
	Women	33,692 (51.4%)	1.57	1.54	1.35	1.77	1.20	1.99	0.31

**Fig. 1:**
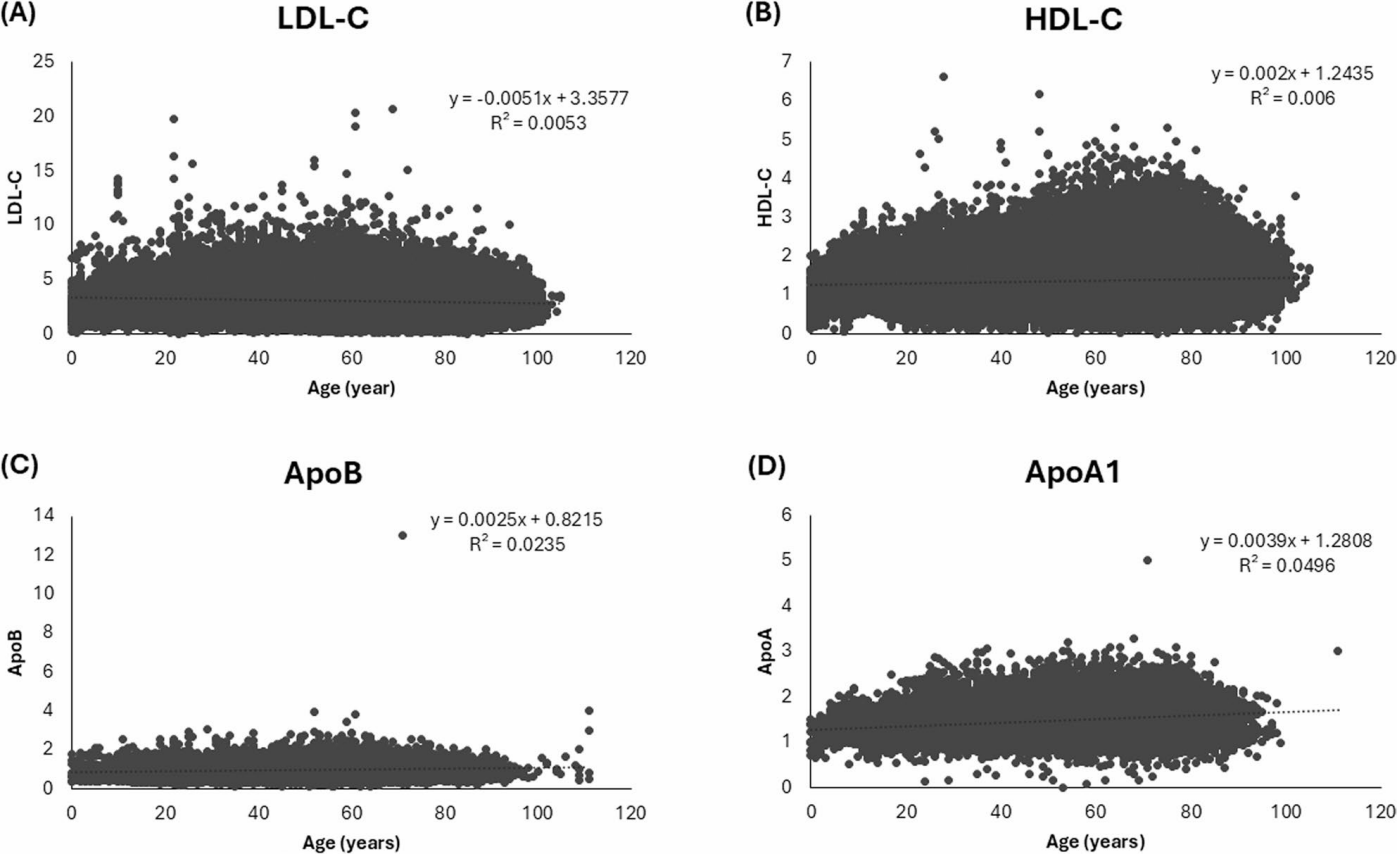
Association of age with plasma/serum concentrations of **(A)** LDL-C, **(B)** HDL-C, **(C)** ApoB, **(D)** ApoA1

We analyzed the association between age and plasma concentrations of ApoB, ApoA1, LDL-C, and HDL-C. Linear regression revealed very weak correlations for all analytes (R² < 0.05), indicating that age does not substantially influence these lipid values in this cohort (see Fig. 1).

### Changes in the number of requests for LDL-C, HDL-C, ApoB, and ApoA1 over time

The number of requested LDL-C analyses has steadily increased from around 2007 to 2024, with a particularly sharp rise in recent years. A similar trend is observed for HDL-C, where the annual number of analyses has grown consistently and reached its highest level in recent years. Both analytes demonstrate a clear, continuous upward trend during this period.

**Fig. 2:**
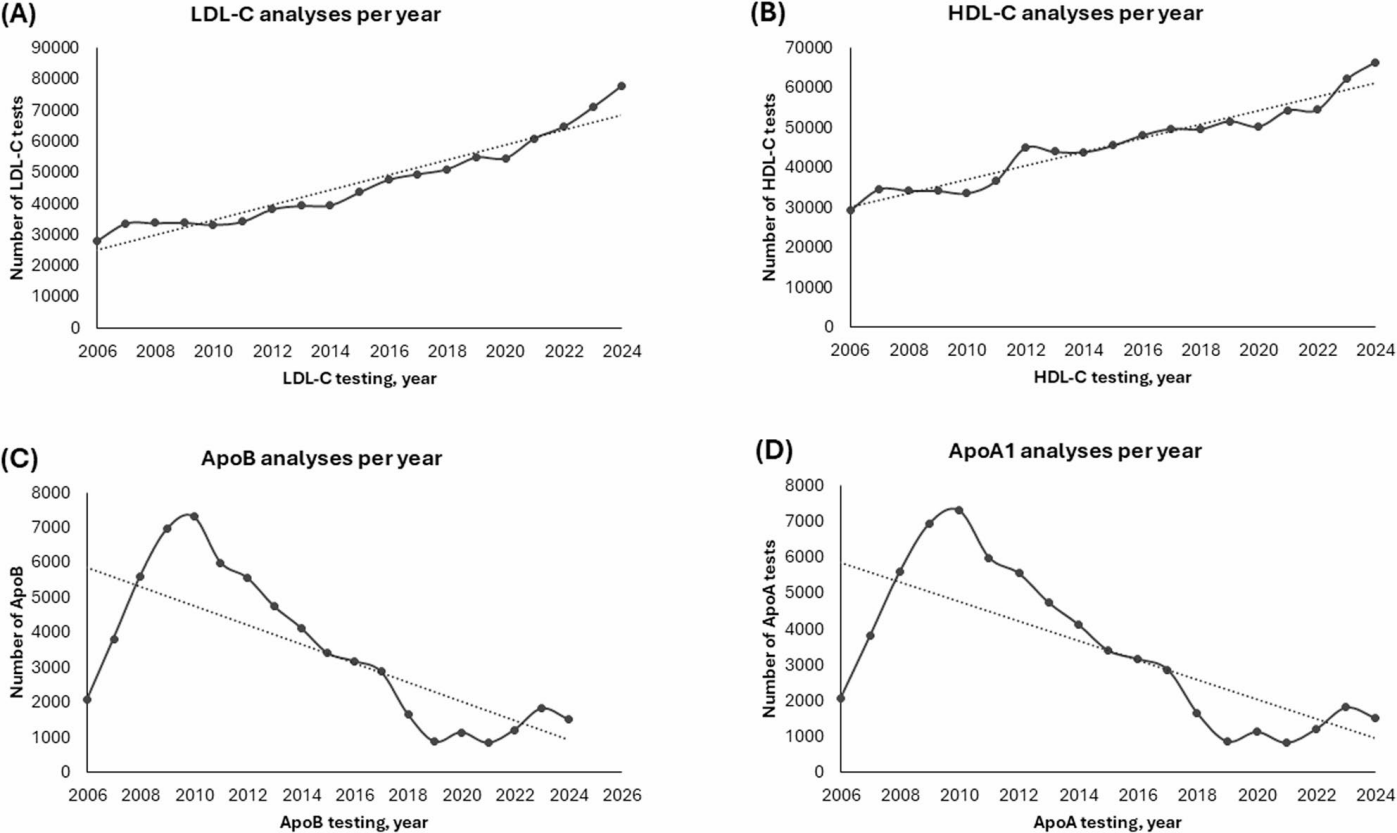
Annual Trends in **(A)** LDL-C, **(B)** HDL-C, **(C)** ApoB, **(D)** ApoA1 tests from 2006 to 2024

In contrast, the number of ApoB and ApoA1 analyses peaked around 2010 and then declined. From 2010 to around 2020, the number of analyses for both ApoB and ApoA1 steadily decreased. After 2020, there was a slight stabilization or minor increase. These trends suggest a change in testing patterns during the observed period (Fig. 2).

### Results of changes in LDL-C, HDL-C, ApoB, and ApoA1 over time

From 2006 to 2024, the median LDL-C concentration decreased from 3.18 to 2.62 mmol/L, suggesting better lipid management among the general population. However, median LDL-C exhibited relatively high variability, with a mean coefficient of variation (CV) of 5.81% and a median CV of 6.36%. These CV values reflect biological variability between individuals over time, as well as analytical variability introduced by different measurement methods, instrumentation, and laboratory settings throughout the study period [16, 17]. This dual contribution to variability is important when interpreting trends and risk associations.

HDL-C levels were relatively stable, with median values ranging from 1.45 to 1.36 mmol/L. The mean coefficient of variation (CV) was 4.21%, and the median CV was 3.96%. These values suggest moderate variability and reflect a combination of intra-individual biological changes and methodological differences [18, 19].

Apolipoprotein B (ApoB) concentrations also showed minimal change, with median values ranging from 0.88 to 0.98 g/L. ApoB had a lower observed variability (mean CV: 2.79%, median CV: 3.16%) compared to LDL-C, suggesting both more consistent biological expression and greater methodological standardization in apolipoprotein testing [13, 16].The median values of ApoA1 fluctuated minimally, ranging from 1.39 to 1.51 g/L. This analyte had the lowest coefficient of variation (CV) of all, with a mean of 2.35% and a median of 2.59%. This low variability indicates good test reproducibility and biological stability, both of which are favorable for long-term risk prediction [17, 20]. The temporal coefficients of variation (CV) for the population medians of LDL-C, HDL-C, ApoB, and ApoA1 over the study period are presented in Table 3.

**Table 3:** The coefficient of variation (CV%) for major blood lipids is as follows: LDL-C, HDL-C, ApoB, and ApoA1, as determined by a summary statistic. These CV values describe Temporal variability in population-level summary statistics over the study period rather than biological variation within individuals or analytical quality control CVs

Summary Statistic	HDL	LDL	ApoB	ApoA1
Mean	4.21	5.81	2.79	2.35
Median	3.96	6.36	3.16	2.59
Lower Quartile	3.87	9.95	3.81	2.45
Upper Quartile	4.23	3.78	2.57	2.59
10th Percentile	4.24	12.91	4.47	2.67
90th Percentile	4.51	2.93	3.02	2.66

The observed systematic shift in lipid values at the method transition point was documented in parallel sample testing and external controls. Medians and trends were annotated by platform in figures, and cross-platform harmonization was incorporated consistently with accepted procedures for accredited Swedish laboratories.

**Fig. 3:**
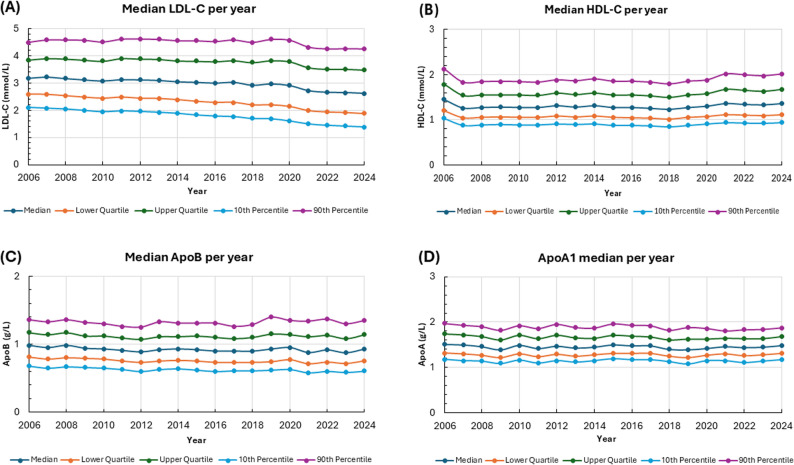
Trends in **(A)** LDL-C, **(B)** HDL-C, **(C)** ApoB, **(D)** ApoA1 Levels by Year (2006–2024) Showing Median, Quartiles, and Percentiles

Statistical and percentile trends revealed a notable decrease in LDL-C levels over the course of the study, while HDL-C levels exhibited a slight increase. In contrast, ApoB and ApoA1 levels remained stable across measured percentiles (Fig. 3; Table 3). These findings reflect absolute changes and relative stability among lipid markers. They provide a foundation for the subsequent discussion of evolving management priorities and the utility of biomarkers in clinical practice.

### Seasonal variation of LDL-C, HDL-C, ApoB, and ApoA1 results

The data reveal minimal seasonal variation across all four analytes. The median values of ApoA1, ApoB, HDL-C, and LDL-C remained stable from January to December, with only subtle changes observed. The lower and upper quartiles, as well as the 10th and 90th percentiles, exhibit similar patterns and do not suggest significant seasonal shifts.

**Fig. 4:**
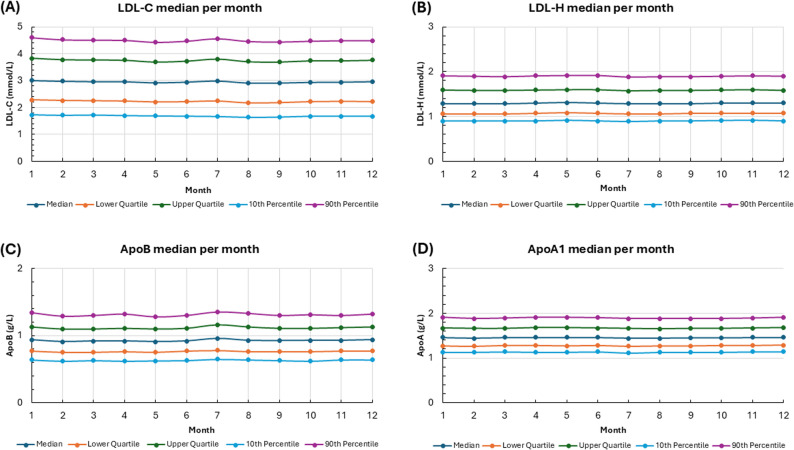
Monthly Variation of **(A)** LDL-C, **(B)** HDL-C, **(C)** ApoB, **(D)** ApoA1 Levels: Median, Quartiles, and Percentiles

The monthly medians and percentile ranges for ApoB and ApoA1 are nearly constant, suggesting biological stability unaffected by season. LDL-C and HDL-C also exhibit stability, with small month-to-month fluctuations that do not suggest a clear seasonal trend (Fig. 4).

**Fig. 5:**
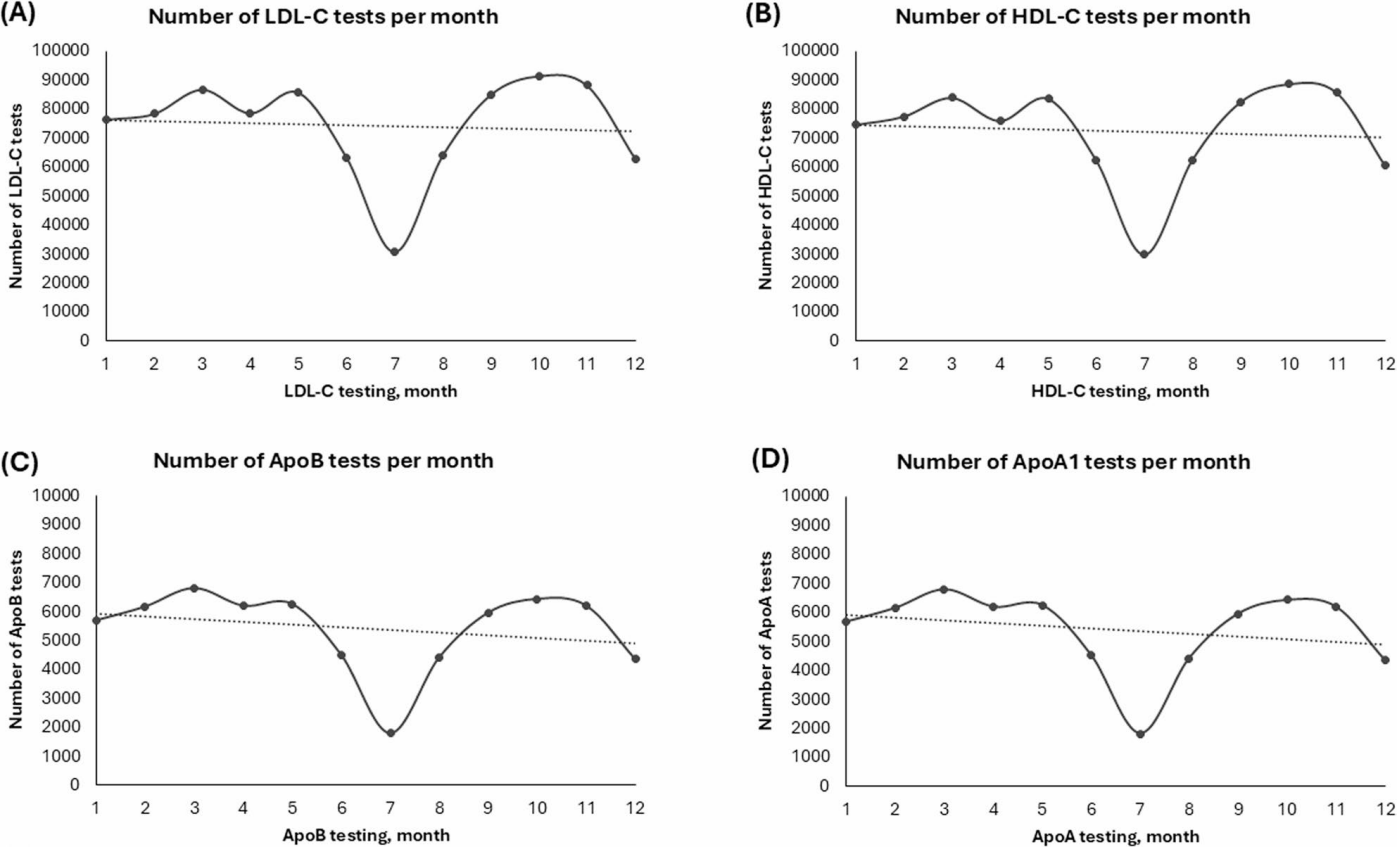
Monthly distribution of laboratory test volumes for **(A)** LDL-C, **(B)** HDL-C, **(C)** ApoB, **(D)** ApoA1

Over the study period, monthly request volumes for LDL-C, HDL-C, ApoB, and ApoA1 analyses exhibited a consistent seasonal pattern. A significant decrease in test requests occurred during the summer months (June–August), with the lowest volumes typically occurring in July. In contrast, test frequency peaked during the spring and fall (Fig. 5).

Despite the significant fluctuations in the number of tests requested, the underlying concentrations of LDL-C, HDL-C, ApoB, and ApoA1 remained stable throughout the year. Analysis of monthly median values, interquartile ranges, and 10th − 90th percentiles revealed minimal variability within the year for all four analytes. There were no systematic peaks or troughs in any month, and lipid concentrations remained biologically consistent across seasons.

## Discussion

Several previous studies have demonstrated seasonal variation in lipid concentrations, with generally higher cholesterol levels in winter and lower levels in summer. This is partially due to physiological and behavioral changes [21, 22]. However, the seasonal amplitude in our population was minimal, consistent with results from other Scandinavian and international cohorts with stable healthcare access [21, 23].

Age-related influences on lipid profiles are well documented, showing increases in total and LDL cholesterol levels through middle age, which sometimes taper off in older adults and are modified by risk reduction efforts [24, 25]. Our finding of weak correlations with age is consistent with modern practices, in which lipid-lowering therapy and screening are common.

Our observation of long-term downward trends in LDL-C, which is supported by international studies in Europe, North America, and the Balkans, underscores the effectiveness of public health initiatives and the increased adoption of lipid management guidelines [26–28]. For instance, Croatian population data shows a significant increase in statin use over 25 years, resulting in substantial reductions in mean lipid values [28].

Since lipid-lowering therapy is typically lifelong, it is crucial that the measurement methods used are stable and reproducible over time with minimal variability. Accuracy at one point in time is not enough – consistency over time matters when decisions about dose adjustments, persistence, and long-term risk assessment depend on small differences in lipid levels.

In our large, population‑based analysis of over 1.8 million lipid and apolipoprotein measurements collected over two decades, we observed notable shifts in laboratory testing, biological variation, and evolving lipid management strategies. The sustained increase in LDL-C and HDL-C testing reflects their pivotal role in cardiovascular risk assessment and ongoing monitoring, as reiterated by the most recent ESC/EAS and ACC/AHA guidelines, which emphasize LDL-C reduction and routine screening for risk evaluation. These recommendations are supported by substantial evidence and are consistent in both European and American clinical practices [3, 29, 30].

The contrasting trends of ApoB and ApoA1 testing – an initial increase followed by a decline – likely reflect the slow adoption and implementation of apolipoprotein guidelines despite their strong predictive value for ASCVD in head-to-head trials and meta-analyses. Recent updates by the European Society of Cardiology (ESC) and the European Atherosclerosis Society (EAS) advocate for ApoB as an adjunct or alternative to LDL-C, especially in complex or high-risk profiles. However, routine integration remains incomplete. The rebound in ApoB/ApoA1 testing observed after 2020 may signal growing clinician awareness and the gradual incorporation of these tests into risk models [3, 4, 31, 32].

The observed reduction in median LDL-C levels, from 3.18 to 2.62 mmol/L, indicates significant improvements in lipid-lowering therapy, increased statin use, and more stringent treatment targets over time. The increase in the number of analyses suggests that more individuals, often with less severe disease, are being tested, which may have contributed to the observed lowering of LDL-C levels. Notably, this trend persisted despite the removal of fasting requirements for lipid testing, which could have otherwise introduced modest upward shifts in LDL-C values due to postprandial variation. The fact that this decline continued despite such changes suggests a true population level improvement in cardiovascular risk management [16].

However, LDL-C demonstrated relatively high visit-to-visit variability, with a mean coefficient of variation (CV) of 5.81%. This variability likely reflects a combination of biological fluctuations (e.g., adherence, diet, and metabolic state) and analytical variability, including differences in laboratory methods, instruments, and sample handling across settings and over time. While some degree of variation is expected in real-world practice, growing evidence shows that high LDL-C variability is independently associated with an increased risk of adverse cardiovascular outcomes, even when mean LDL-C remains within the target range [18, 19, 33, 34]. Accordingly, recent guidelines emphasize not only reducing LDL-C but also achieving stable lipid control through consistent monitoring, medication adherence, and making appropriate therapeutic adjustments [31].

In contrast, ApoB and ApoA1 showed much lower variability (CVs below 3.2%). This likely reflects the greater stability and reproducibility of apolipoprotein assays. In particular, ApoB benefits from standardized, mass-based measurement techniques that are less influenced by fasting status, triglyceride levels, or estimation formulas (e.g., the Friedewald equation) [17, 20]. These properties make ApoB reliable for long-term monitoring of atherogenic lipoprotein burden. Consequently, ApoB has been shown to outperform LDL-C in predicting cardiovascular risk, particularly in patients with metabolic syndrome, diabetes, or discordant lipid profiles [35, 36].

The combination of low variability and strong predictive value makes apolipoproteins attractive for longitudinal risk assessment. However, their stability must be interpreted in context: lower variability does not automatically mean better predictive power unless supported by outcome data. Nevertheless, their reduced fluctuation may offer an operational advantage by minimizing misclassification due to biological or technical noise, particularly in high-risk or diagnostically complex populations [37].

Finally, the relative stability of HDL-C and ApoA1 in both absolute levels and variability may reflect the plateauing of public health interventions targeting lifestyle-related factors. Nevertheless, the modest upward trend in HDL-C observed in our data could indicate ongoing improvements in physical activity, diet, or the use of combination lipid-lowering therapies [38].

Notably, the absence of clinically meaningful seasonal variation in lipid or apolipoprotein concentrations reinforces the validity of using single-point measurements for cardiovascular risk stratification throughout the year, as recommended by guidelines. Seasonal dips in test request volumes reflect operational changes within healthcare systems rather than true physiological variation. This finding is robust across subgroups and consistent with prior report [21, 39].

Sex differences were evident: females had higher HDL-C and ApoA1 values, slightly higher LDL-C, and similar ApoB levels compared to males. These patterns mirror the established physiology of lipids, which is driven by hormonal and metabolic factors. This finding reinforces the potential utility of ApoB as a sex-neutral marker of atherogenic burden. Age-related correlations with lipid measures were weak (R² < 0.05), possibly due to effective clinical control or the masking effect of treatment in older populations [4, 32]. At very low LDL-C concentrations, the relative CV becomes disproportionately large in relation to the absolute value, which can inflate the calculated reference change value. This should be taken into account when interpreting serial LDL-C measurements in patients with very low concentrations.

Since our study is based on a retrospective dataset, median patient monitoring was not used as part of the real-time quality assurance process during the study period. Instead, we analyzed medians retrospectively to demonstrate their potential for detecting assay shifts that standard quality control procedures may not always capture. Our results show that under certain conditions, tracking patient-derived medians can help identify trends or shifts in assay performance that routine QC data alone may not reveal. While this study did not implement median monitoring as an active QC measure, future studies could evaluate the added value of integrating median-based monitoring prospectively as a supplementary alert system for long-term analytical stability in accredited laboratories.

Our results represent a large-scale descriptive evaluation of lipid and apolipoprotein trends in routine laboratory practice. The relationship between patient medians, analytical stability, and cardiovascular risk prediction must be tested in outcome-based studies. These findings should inform future research on integrating laboratory results with clinical and treatment outcomes.

### Strengths and limitations

The major strengths of this study include its large sample size, extended observation period spanning nearly two decades, and use of accredited, standardized laboratory methods. These factors collectively enhance the robustness and generalizability of the findings. Including both male and female patients across a broad age range increases the relevance of the results to diverse clinical populations.

However, several limitations warrant consideration. The lack of detailed clinical and outcome data restricts the ability to adjust for potential confounders, such as medication use, comorbidities, and lifestyle factors. Additionally, the exact fasting status was not documented for all patients. This may contribute to residual variability and limit the interpretation of absolute lipid levels. This limitation also restricts the assessment of direct clinical implications or risk associations. Methodological changes over time, including transitions between analytical platforms and the use of serum and plasma samples, may introduce biases that affect comparability. Additionally, since the study was conducted at a single center, the findings may be applicable only to that specific population or healthcare setting. These factors should be carefully considered when interpreting the results.

## Conclusions

This two-decade analysis of over 1.8 million lipid and apolipoprotein measurements shows that population-level median patient values are a useful supplementary tool for monitoring laboratory performance and identifying long-term trends in lipid management. The decline in LDL-C and the stable or modestly improved HDL-C levels suggest the successful implementation of cardiovascular prevention strategies. The relatively low variability of ApoB and ApoA1 indicates their analytical and biological stability. This reinforces their role in long-term risk assessment, particularly in complex lipid profiles.

The absence of significant seasonal variation in sex-specific differences underscores the reliability of year-round lipid testing and highlights the importance of sex-neutral markers, such as ApoB. The increasing test volumes for LDL-C and HDL-C reflect their established role in guidelines, while the fluctuating adoption of apolipoproteins illustrates their gradual integration into practice.

Overall, these findings support incorporating median patient values into routine quality assurance programs and reaffirm the clinical relevance of traditional lipids and apolipoproteins in cardiovascular risk stratification. However, future studies linking laboratory data with clinical outcomes and treatment patterns are needed to clarify the prognostic impact of lipid variability and guide more individualized prevention strategies.

## Data Availability

Data are available from the corresponding author upon reasonable request.

## Abbreviations

ACC: American College of Cardiology
AHA: American Heart Association
ApoA1: Apolipoprotein A1
ApoB: Apolipoprotein B
ASCVD: Atherosclerotic Cardiovascular Disease
CVD: Cardiovascular Disease
CV: Coefficient of Variation
CV%: Coefficient of Variation (expressed as a percentage)
EAS: European Atherosclerosis Society
EDTA: Ethylenediaminetetraacetic acid
ESC: European Society of Cardiology
HDL-C: High-Density Lipoprotein Cholesterol
IQR: Interquartile Range
LDL-C: Low-Density Lipoprotein Cholesterol
QC: Quality control
mmol/L: Millimoles per Liter
g/L: Grams per Liter
